# Anthocyanins Isolated from *Vitis coignetiae Pulliat* Enhances Cisplatin Sensitivity in MCF-7 Human Breast Cancer Cells through Inhibition of Akt and NF-κB Activation

**DOI:** 10.3390/molecules25163623

**Published:** 2020-08-09

**Authors:** Anjugam Paramanantham, Min Jeong Kim, Eun Joo Jung, Hye Jung Kim, Seong-Hwan Chang, Jin-Myung Jung, Soon Chan Hong, Sung Chul Shin, Gon Sup Kim, Won Sup Lee

**Affiliations:** 1 Departments of Internal Medicine, Institute of Health Sciences, Gyeongsang National University Hospital, Gyeongsang National University School of Medicine, Jinju 660-702, Korea; anju.udhay@gmail.com (A.P.); bokdae@hanmail.net (M.J.K.); 2 Research Institute of Life Science and College of Veterinary Medicine, Gyeongsang National University, 501 Jinju-daero, Jinju 52828, Korea; 3 Departments of Biochemistry Institute of Health Sciences, Gyeongsang National University Hospital, Gyeongsang National University School of Medicine, Jinju 660-702, Korea; eunjoojung@gnu.ac.kr; 4 Department of Pharmacology, College of Medicine, Institute of Health Sciences Gyeongsang National University School of Medicine, Jinju 660-702, Korea; hyejungkim@gnu.ac.kr; 5 Department of Surgery, Konkuk University School of Medicine, Seoul 05030, Korea; csh@kuh.ac.kr; 6 Departments of Neurosurgery, Institute of Health Sciences and Gyeongsang National University Hospital, Gyeongsang National University College of Medicine, 90 Chilam-dong, Jinju 660-702, Korea; gnuhjjm@gnu.ac.kr; 7 Departments of Surgery, Institute of Health Sciences and Gyeongsang National University Hospital, Gyeongsang National University College of Medicine, 90 Chilam-dong, Jinju 660-702, Korea; hongsc@gnu.ac.kr; 8 Department of Chemistry, Research Institute of Life Science, Gyeongsang National University, Jinju 660-701, Korea; scshin@gsnu.ac.kr

**Keywords:** synergistic effects, CDDP, meoru, phytochemicals, AIMs, cisplatin resistance

## Abstract

Anthocyanins isolated from *Vitis coignetiae Pulliat* (Meoru in Korea) (AIMs) have various anti-cancer properties by inhibiting Akt and NF-κB which are involved in drug resistance. Cisplatin (CDDP) is one of the popular anti-cancer agents. Studies reported that MCF-7 human breast cancer cells have high resistance to CDDP compared to other breast cancer cell lines. In this study, we confirmed CDDP resistance of MCF-7 cells and tested whether AIMs can overcome CDDP resistance of MCF-7 cells. Cell viability assay revealed that MCF-7 cells were more resistant to CDDP treatment than MDA-MB-231 breast cancer cells exhibiting aggressive and high cancer stem cell phenotype. AIMs significantly augmented the efficacy of CDDP with synergistic effects on MCF-7 cells. Molecularly, Western blot analysis revealed that CDDP strongly increased Akt and moderately reduced p-NF-κB and p-IκB and that AIMs inhibited CDDP-induced Akt activation, and augmented CDDP-induced reduction of p-NF-κB and p-IκB in MCF-7 cells. In addition, AIMs significantly downregulated an anti-apoptotic protein, XIAP, and augmented PARP-1 cleavage in CDDP-treated MCF-7 cells. Moreover, under TNF-α treatment, AIMs augmented CDDP efficacy with inhibition of NF-κB activation on MCF-7 cells. In conclusion, AIMs enhanced CDDP sensitivity by inhibiting Akt and NF-κB activity of MCF-7 cells that show relative intrinsic CDDP resistance.

## 1. Introduction

Breast cancer is one of the most frequently occurring cancers and 1 in 4 diagnosed cancer cases for women had breast cancer in 2018 [1]. Recent studies have shown that the rate of cancer incidence has declined. However, breast cancer continues to be the reason for the high fatality rate in females, especially between the ages of 34–65 in many countries including South Korea [2]. The main reason for high fatality is the treatment failure due to drug resistance [3]. Many researchers believe one possible way to overcome or delay the development of resistance is the combined administration of drugs with non-overlapping mechanisms of action, which is a combination chemotherapy. However, this combination approach with multiple chemotherapeutic agents leads to serious toxicities even to death. Hence, recent solutions to drug resistance are generally based on the addition of novel targeted drugs or non-toxic drugs that increase efficacy or previous drug sensitivity by identification of cancer cell dependencies and/or the resistance mechanisms [4].

Cisplatin (cis-diaminedichloroplatinum, CDDP) has been one of the most widely used effective chemotherapeutic agents for more than 30 years. CDDP is a DNA damaging drug that induces inhibition of DNA synthesis and/or RNA transcription, and also cell cycle arrest [4,5,6]. CDDP resistance is associated with various mechanisms such as deregulated MAPK or PI3K/Akt pathway [7], increased tolerance of DNA damage [8], anti-apoptotic proteins [9], or suppressed apoptotic activities [10].

Phytochemicals showed anti-cancer effects by safely modulating tumor suppressor genes or the signaling related to cancer cell survival or death [11]. Many interests have been drawn in cancer prevention and treatment. However, there are only some trials to overcome drug resistance or to increase chemotherapy efficacy by these phytochemicals. We here consider how phytochemicals can be integrated to prevent, delay, or revert resistance to chemotherapy. Hence, as an initial step, we tested whether phytochemicals increased chemotherapy efficacies by inhibiting a well-known signaling mechanism involved in CDDP resistance events through various mechanisms were reported for CDDP resistance.

Recently, many studies reported that the anthocyanins have anti-cancer, anti-inflammatory, and angiogenesis activity [12,13,14]. We reported that anthocyanins isolated from *Vitis coignetiae Pulliat* (Meoru in Korea) (AIMs) have various anti-cancer properties and promotes apoptosis by inhibiting Akt and NF-κB [15,16]. Activation of Akt and NF-κB is one of the CDDP resistance mechanisms [17]. In addition, there are many studies to overcome the drug resistance by targeting NF-κB or Akt [18,19]. Breast cancer is one of the most common causes of cancer mortality in women [20]. Literature studies reported that MCF-7 human breast cancer cells have high resistance to CDDP compared to other breast cancer cell lines; the IC50 value of MCF-7 cells to CDDP was found to be 97 µM, whereas that of MDA-MB-231 breast cancer cells that show aggressive and high cancer stem cell phenotypes were 36 µM [21]. In addition, MCF-7 cells also have a defect in inducing caspase-mediated apoptosis because of defect in caspase 3 expression [22]. In this study, we postulated that the AIMs can enhance the effect of CDDP by the inhibition of NF-κB and Akt signaling on MCF-7 cells that showed intrinsic CDDP resistance. Hence, we investigated the anti-cancer effects of AIMs on CDDP-treated MCF-7 cells that show relative intrinsic CDDP resistance, and their underlying cellular mechanisms.

## 2. Results

### 2.1. MCF-7 Cells Were More Resistant to CDDP Than MDA-MB-231 Cells, and Anthocyanins Isolated from Vitis coignetiae Pulliat (AIMs) Induced Anti-Proliferative Effects

**Figure 1:**
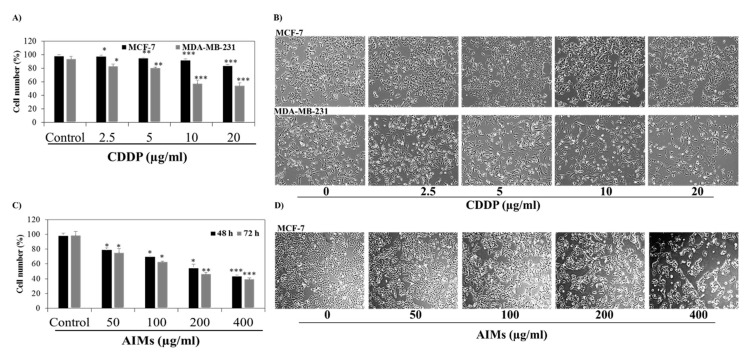
The inhibitory effects of CDDP and AIMs on breast cancer cell lines. (**A**) trypan blue exclusion assay to analyze the CDDP sensitivity of MCF-7 and MDA-MB-231 cells. Cells were treated with a concentration of 0, 2.5, 5, 10, and 20 µg/mL of CDDP, and trypan blue assay was performed 48 h after CDDP treatment. MCF-7 cells showed relative resistance to CDDP and MDA-MB-231 cells showed inhibition of cell proliferation in a dose dependent manner; (**B**) morphological representation of MCF-7 and MDA-MB-231 cells under a light microscope. Cells were treated with CDDP at different concentrations (0, 2.5, 5, 10, and 20 µg/mL) for 48 h; (**C**) trypan blue exclusion assay for AIMs sensitivity of MCF-7 cells. Cells were treated with an indicated of AIMs for 48 and 72 h; (**D**) morphological representation of MCF-7 cells under the light microscope. Cells were treated with AIMs at different concentrations (0, 50, 100, 200, and 400 µg/mL) for 48 and 72 h showed inhibitory effects in a dose dependent manner. All data shown are the mean ± SD of three different experiments performed independently. * *p* < 0.05, ** *p* < 0.01 and *** *p* < 0.0001 between untreated control and treated groups.

To evaluate the effect of CDDP on human breast cancer cell lines, we treated different concentrations of CDDP (0, 2.5, 5, 10, and, 20 µg/mL) in both MCF-7 and MDA-MB-231 cells for 48 h. Trypan blue exclusion assay revealed that CDDP had far less effects on MCF-7 cells than on MDA-MB-231 cells. The morphological analysis also divulged that cell proliferation of MDA-MB-231 cells was greatly inhibited compared to that of MCF-7 cells in treatment with CDDP (Figure 1A). These results suggest that MCF-7 cells are resistant to CDDP treatment. Trypan blue assay clearly revealed that AIMs inhibit cell viability in a dose-dependent manner in MCF-7 cells. MCF-7 cells treated with AIMs at the concentration of 400 µg/mL showed 46% and 42% cell viability at 48 h and 72 h, respectively (Figure 1C). Furthermore, a microscopic observation also showed suppression of cell proliferation and some cell death (Figure 1D). These results indicate that AIMs alone mainly produced anti-proliferative effects on MCF-7 cells. 

### 2.2. AIMs Induced a Synergistic Effect on Cell Death of MCF-7 Cells with Co-Treatment of CDDP

**Figure 2:**
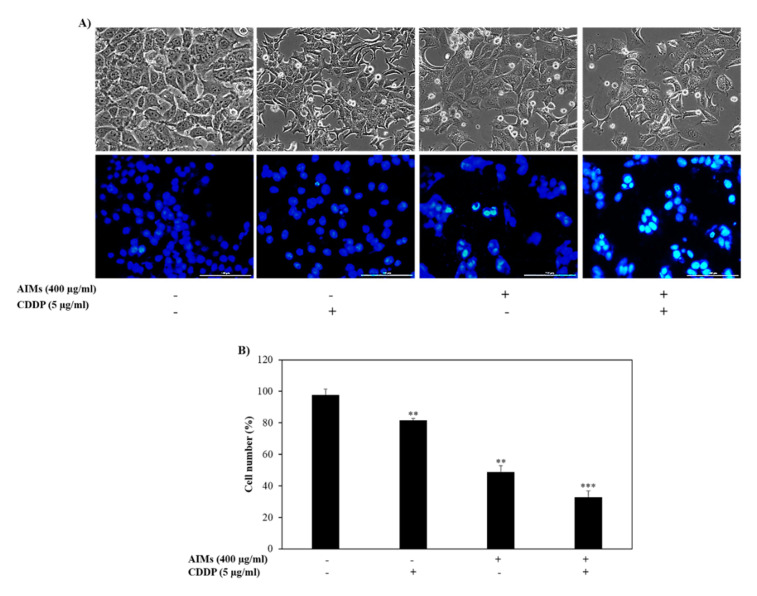
The inhibitory effects of anthocyanins isolated from *Vitis coignetiae Pulliat* (anthocyanins isolated from meoru, AIMs) and/or cis-diaminedichloroplatinum (CDDP) on MCF-7 cells human breast cancer cells. The cells were treated with 400 µg/mL of AIMs one hour before 5 µg/mL of CDDP treatment and incubated for 48 h. (**A**) After 48 h of treatment, pictures were taken representing cellular and nuclear morphology of the cells which showed damages cells and decreased cell proliferation. DAPI staining was used to show the deformed nucleus. (**B**) The inhibitory effects of combined AIMs and CDDP were analyzed through the trypan blue exclusion assay. “+” and “−” represents the presence and absence of the compound, absence of the compound specified. The data shown are mean ± SD of three different experiments performed separately. ** *p* < 0.05 versus treated group and *** *p* < 0.05 versus CDDP treated group.

MCF-7 cells are relatively resistant to CDDP as compared to other breast cancer cell lines (Figure 1A) [23]. MCF-7 cells that were treated with AIMs combined with CDDP showed a high number of cell death at 48 h. Morphological analysis through a phase contrast microscope also revealed an increase in cell death and deformed cells with the combined treatment of AIMs and CDDP. To evaluate the type of cell death with DAPI staining, MCF-7 cells clearly displayed condensed and fragmented nuclei, the hallmark cell morphology of apoptosis (Figure 2A). In addition, the MTT assay revealed that there is a synergetic effect between CDDP and AIMs on cell viability of MCF-7 cells. The synergistic effect is calculated as described previously [24]; the viability of cells treated with CDDP alone, AIMs alone, and a combination of CDDP and AIMs group were 81%, 58%, and 37%, respectively (Figure 2B). These results strongly suggest that AIMs may aid MCF-7 cells to overcome CDDP resistance.

### 2.3. AIMs Enhanced CDDP Efficacy in MCF-7 Cells That Showed Relative CDDP Resistance

**Figure 3:**
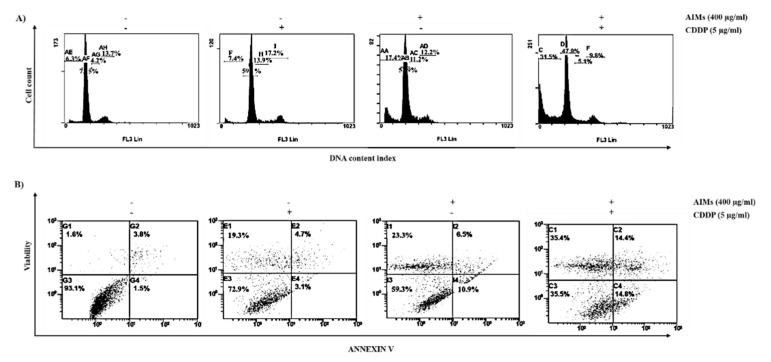
Synergistic effects between AIMs and CDDP on cell death of MCF-7 cells. MCF-7 cells were treated with 400 μg/mL of AIMs 1 h before 5 μg/mL of CDDP treatment and incubated for 48 h (**A**) Flow cytometry for cell cycle analysis. MCF-7 cells were stained with PI (Propidium Iodide) and subjected to flow cytometry analysis. The population of the cells in subG1 phase has been highly increased when AIMs and CDDP were treated together (**B**) flow cytometry with Annexin V/PI double staining. MCF-7 cells were treated with the given treatment (400 µg/mL of AIMs and 5 μg/mL of CDDP for 48 h) and stained with Annexin V/PI to analyze through flow cytometry. The number of cells in the fourth quadrant increased significantly showing high cell death when AIMs and CDDP treated together. “+” and “−“represents the presence and absence of the compound, absence of the compound specified.

To explore whether AIMs co-treated with CDDP augments CDDP-induced cell cycle arrest or cell death of MCF-7 cells, we evaluate the distribution of cell cycle by flow cytometer. PI staining depicts that a combination treatment with AIMs and CDDP showed a significant increase in the cell populations in the sub-G1 phase. These results indicated that there was a synergistic effect between AIMs and CDDP on MCF-7 cells; the percentage of cells in the sub-G1 phase of control, CDDP alone, AIMs alone, and combination of CDDP and AIMs group were 6.3%, 7.4%, 17.4%, and 31.5%, respectively (Figure 3A). Furthermore, Annexin V/PI staining also showed an increasing number of dead cells when AIMs and CDDP were treated together. The number of dead cells in the 4th quadrant drastically increased from 1.6% to 35.4% when AIMs were treated 1 h before CDDP treatment (Figure 3B). Taken together, these results signify that AIMs enhanced CDDP efficacy in MCF-7 cells that showed relative CDDP resistance.

### 2.4. AIMs Enhanced CDDP Efficacy by Inhibiting NF-κB and Akt Activation in MCF-7 Cells That Showed Relative CDDP Resistance

**Figure 4:**
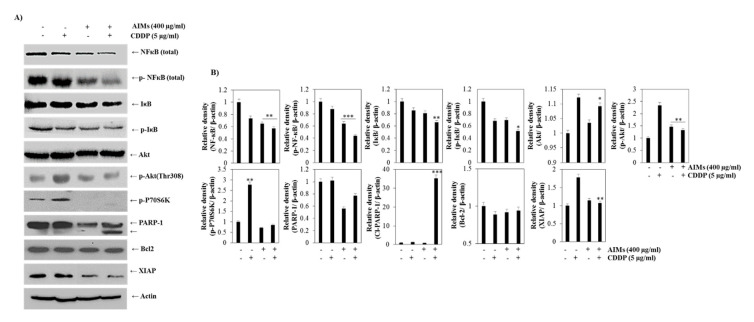
The effect of CDDP with or without AIMs on NF-κB activity in MCF-7 cells. The proteins were isolated from MCF-7 cells treated with 400 µg/mL of AIMs and 5 µg/mL of CDDP for 48h. (**A**) Equal amounts of cell lysate (30 μg) were resolved by SDS polyacrylamide gels and transferred onto nitrocellulose membranes. The membranes were probed with the indicated antibodies and detected by the enhanced chemiluminescence detection system. Western blot analysis for Akt, NF-κB and NF-κB regulated proteins. The data shown here are representative of at least three independent experiments. (**B**) Densitometry analysis of Western blot bands. The values were normalized against actin and expressed as a mean of ± SD of at least three independent experiments * *p* < 0.05, ** *p* < 0.01, and *** *p* < 0.0001 between control and treated groups. “+” and “−” represents the presence and absence of the compound, absence of the compound specified.

MCF-7 cells acquire CDDP resistance through activating numerous pathways which include protein kinase activation such as Akt and PKC (protein kinase C) [21]. It is reported that CDDP induced a more marked decrease in p-IκB and NF-κB activity in CDDP-sensitive cells than in CDDP-resistant cells [25]. Western blot analysis revealed that CDDP, consistent with the previous finding, strongly increased Akt and moderately reduced p-NF-κB and p-IκB (Figure 4A) and that AIMs inhibited CDDP-induced Akt activation, and augmented CDDP-induced reduction of p-NF-κB and p-IκB in MCF-7 cells. In addition, AIMs significantly downregulated an anti-apoptotic protein, XIAP and augmented PARP-1 cleavage when treated with CDDP (Figure 4). These results suggest that AIMs, when treated with CDDP, could promote cell death of MCF-7 cells by inhibition of Akt phosphorylation and NF-κB activation followed by inhibition of XIAP.

### 2.5. TNF-α Enhanced the CDDP Sensitivity of Both MCF-7 and MDA-MB-231 Cells, But the Intensity Was Different between Them; MCF-7 Cells Are Still Less Sensitive to the Combination Treatment of TNF-α and CDDP

**Figure 5:**
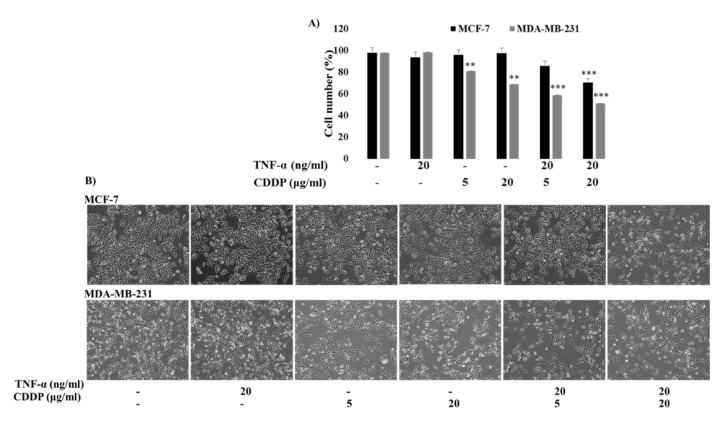
The effect of combination treatment of TNF-α and CDDP on MCF-7 and MDA-MB-231 cells. (**A**) The cell viability is measured by the trypan blue exclusion assay. MCF-7 and MDA-MB-231 cells were pre-treated with 20 ng/mL of TNF-α 1 h at 37 °C followed by the treatment of 5 and 20 µg/mL of CDDP. The cells were stained with trypan blue and counted using a haemocytometer and percentage of viable cells were calculated. (**B**) The morphological representation of TNF-α treated MCF-7 and MDA-MB-231 cells with CDDP. The cells were pre-treated with 20 ng/mL of TNF-α 1 h at 37 °C followed by the treatment of 5 and 20 µg/mL of CDDP. The data shown are mean ± SD of three different experiments performed separately. ** *p* < 0.05 versus treated group and *** *p* < 0.05 versus CDDP treated group. “−” represents the absence of the compound, absence of the compound specified.

It was reported that TNF-α increased CDDP sensitivity of cancer cells [26]. We tested whether TNF-α augmented CDDP sensitivity of both MCF-7 and MDA-MB-231 cells because it was reported that the two cells showed different sensitivity to TNF-α [27]; MCF-7 cells were responsive to TNF-α treatment while MDA-MB-231 cells are resistant to it. As shown in Figure 5, TNF-α increased CDDP sensitivity of both the cancer cells, but there was a difference in intensity; an additive effect and synergistic effect were observed between CDDP and TNF-α in MCF-7 cells and MDA-MB-231 cells, respectively. This finding suggests that MCF-7 cells were still less sensitive to CDDP and TNF-α treatment even though MCF-7 cells did not show resistance to TNF-α alone.

### 2.6. AIMs Significantly Enhanced the Effects of TNF-α Alone and Combination Treatment of TNF-α and CDDP

**Figure 6:**
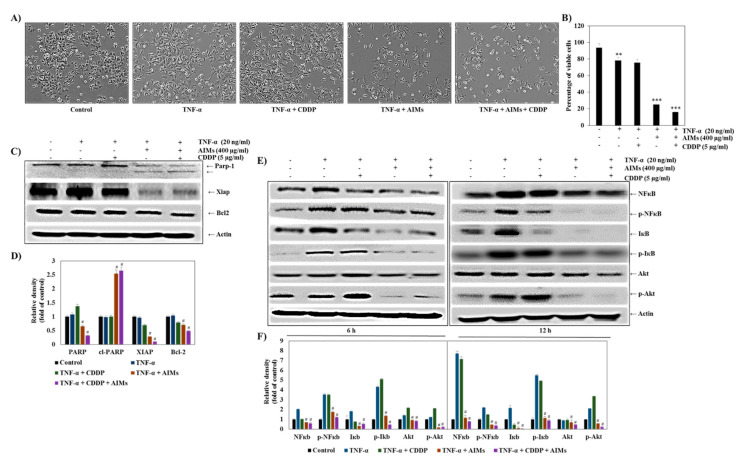
The inhibitory effects of AIMs on MCF-7 cells treated with TNF-α and CDDP treatment. (**A**) the morphological representation of TNF-α treated cells with AIMs and CDDP. MCF-7 cells were pretreated with 400 µg/mL of AIMs 1 h at 37 °C, subsequently treated with or without 5 µg/mL of CDDP and 20 ng/mL of TNF-α at 37 °C, incubated for 48 h. (**B**) The cell viability is measured by the trypan blue exclusion assay. The data shown are mean ± SD of three different experiments performed separately. ** *p* < 0.05 non-treated versus treated group and *** *p* < 0.05 TNF-α treated versus AIMs and CDDP treated group. (**C**) Western blot analysis for cytotoxic effect of TNF-α, AIMs, and CDDP combined treatment for 48 h. The total lysates of MCF-7 cells with the above-mentioned treatment were resolved on SDS-polyacrylamide gels followed by transfer to PVDF membrane and probed with the specific primary and secondary antibody. The protein was visualized using chemidoc with the ECL detection kit. The data shown here are representative of at least three independent experiments. (**D**) The densitometry analysis of Western blot bands was normalized against actin and expressed as a mean of ± SD of at least three independent experiments # *p* < 0.05 TNF-α treated versus AIMs and CDDP treated group; (**E**) Western blot analysis of time dependent TNF-α treatment. MCF-7 cells were treated with 400 AIMs µg/mL of AIMs for1 h at 37 °C, subsequently treated with or without 5 µg/mL of CDDP and 20 ng/ml of TNF-α for 6 h and 12 h at 37 °C. The total lysates of MCF-7 cells with the above-mentioned treatment were resolved on SDS-polyacrylamide gels followed by transfer to PVDF membrane and probed with the specific primary and secondary antibody. The protein was visualized using chemidoc with the ECL detection kit. The data shown here are representative of at least two independent experiments. (**F**) The densitometry analysis of Western blot bands was normalized against actin and expressed as a mean of ± SD of at least three independent experiments # *p* < 0.05 TNF-α treated versus AIMs and the CDDP treated group. “+” and “−” represents the presence and absence of the compound, absence of the compound specified.

**Figure 7:**
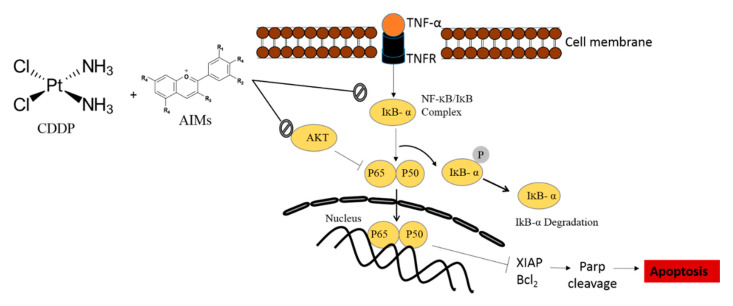
The schematic diagram representing AIMs potentiating CDDP sensitivity through NF-κB inhibition. In this study, CDDP resistance of MCF-7 cells was at least in part involved in Akt activation and NF-κB activation; CDDP significantly increased p-Akt and followed by maintaining p-NF-κB, and p-IκB which are usually suppressed by CDDP in CDDP-sensitive cancer cells [15]. AIMs inhibited CDDP-induced Akt activation and suppressed maintained NF-κB activity. Through these two mechanisms, AIMs enhanced CDDP efficacy of MCF-7 cells that showed relative resistance by X-linked inhibitor of apoptosis protein (XIAP). AIMs contains the following composition; delphinidin-3,5-diglucoside:cyanidin-3,5-diglucoside:petunidin-3,5-diglucoside:delphinidin-3-glucoside:malvdin-3,5-diglucoside:peonidin-3,5-diglucoside: cyanidin-3-glucoside:petunidin-3-glucoside:peonidin-3- glucoside:malvidin-3-glucoside ¼ 1.0:0.5:3.4:28.1:6.4:6.4:4.2: 2.5:4.9:22.5:5.0:22.6.

Then, we assessed the effects of AIMs on MCF-7 cells treated with TNF-α and CDDP. AIMs significantly suppressed cell viability of MCF-7 cells treated with TNF-α alone and TNF-α and CDDP together (Figure 6A). There were synergistic effects between AIMs and TNF-α alone or TNF-α and CDDP; AIMs with TNF-α alone and with TNF-α and CDDP in combination induced 25% and 16% cell viability of MCF-7 cells, respectively (Figure 6B). This finding suggests that AIMs enhances anti-cancer effects by inhibiting NF-κB activation because TNF-α is a known NF-κB stimulant and its anti-cancer effects were not clearly observed by NF-κB activation [28]. To confirm this finding at molecular level, we performed Western blot analysis. It revealed that 6 h and 12 h treatment of TNF-α significantly induced NF-κB activation, and its effect was inhibited by AIMs in MCF-7 cells (Figure 6E). These findings suggested that AIMs ameliorate CDDP efficacy by inhibiting NF-κB activation in MCF-7 cells. Figure 7 shows the schematic diagram of the AIMs and the CDDP effect on MCF-7 breast cancer cells.

## 3. Discussion

In this study, we postulated that the AIMs can enhance the effect of CDDP by the inhibition of NF-κB and Akt signaling on MCF-7 cells that showed intrinsic CDDP resistance. We found that AIMs significantly enhance the efficacy of CDDP on MCF-7 cells by inhibiting CDDP-induced Akt activation and maintained NF-κB activity while on CDDP treatment. It was reported that CDDP inhibits significantly NF-κB activity by suppressing p-IκB in CDDP-sensitive cancer cells and that an inhibitor of NF-κB that blocks IκB phosphorylation showed overcome relative CDDP resistance, even though CDDP usually suppresses NF-κB activity in cancer cells [19,29].

We recently demonstrated that AIMs served as an inhibitor of NF-κB in MCF-7 cells [28]. NF-κB activation involved in drug resistance by suppressing pro-apoptotic and inducing anti-apoptotic molecules [30,31]. Most of the previous studies of AIMs showed predominantly anti-cancer activity by inhibiting NF-κB and NF-κB-regulated proteins involved in apoptosis, angiogenesis, and metastasis [28,32]. In addition, there are reports that CDDP resistance was reversed by an NF-KB inhibitor; in that study, an NF-κB inhibitor, BAY 117,085 augmented CDDP effects by suppressing the expression of XIAP. This finding is consistent with our result. In addition, a soy isoflavone, genistein, increased CDDP sensitivity by inhibiting NF-κB activity in pancreatic cancer cells [33]. Furthermore, evidence suggests that NF-κB and Akt activation is one of the major causes to attain CDDP resistance in cancer cells [31]. These findings all support that AIMs could enhance the efficacy of CDDP on MCF-7 cells by inhibiting NF-κB and an NF-κB-regulated protein, XIAP.

However, the NF-κB signaling cascade is also one of the downstream signals of the PI3K/AKT pathway, activation of Akt promotes IκB degradation via phosphorylating IKKα kinase [17]. In MCF-7 cells, some may think that inhibition of Akt would be the main mechanism for boosting CDDP efficacy because AIMs clearly inhibited the activation of Akt that was induced by CDDP (Figure 4) and that Akt regulates NF-κB by suppressing IκB degradation and also promotes cancer cell survival by activating anti-apoptotic proteins and inactivating pro-apoptotic proteins [34]. We also agreed in some part because AIMs showed anti-cancer effects by inhibition of Akt activity [35]. However, with AIMs effects on Akt, it is hard to explain the synergistic effect between TNF-α and CDDP; TNF-α in combination with AIMs with or without CDDP exhibited a remarkable anti-cancer effect with high efficacy on reducing cell viability (Figure 6). In addition, cancer cells with no NF-κB activity showed high sensitivity to TNF-α treatment and other chemotherapeutic drugs [36]. These findings support that AIM effects on CDDP also should be attributed to inhibition of NF-κB at least in part.

Regarding chemotherapeutic strategy, many researchers were interested in NF-κB inhibitors rather than Akt inhibitors even though the direct cause of CDDP resistance appeared to be up-regulation of Akt [18,19]. The reason would be the toxicities of Akt inhibitors; Akt signaling plays a critical role in maintaining normal cell homeostasis. As previously shown, AIMs 400 µg/ml of AIMs did not show toxicity to normal cells [28]. These findings suggest that AIMs inhibit Akt signaling indirectly, probably upstream target molecules. As supporting evidence, a certain anthocyanin directly binds to EGFR and AIMs also inhibit EGFR and non-EGFR related Akt signaling [37]. This finding suggests that only under EGFR high expression do anthocyanins serve as a signaling inhibitor and that the toxicity would be insignificant. In addition, most previous studies of AIMs showed predominantly anti-metastatic activity by inhibiting Akt or NF-κB and its downstream molecules with moderate inhibition of cell proliferation rather than cell killing [32]. These findings suggest that the addition of AIMs to CDDP treatment is a safe strategy to enhance CDDP efficacy on cancer cells.

The limitations of the study are as follows. First, this study did not clearly demonstrate that how AIMs inhibit Akt or NF-κB signaling regarding the enhancing effects of AIMs on CDDP efficacy of MCF-7 cells as well as which signaling is more important in enhancing CDDP efficacy. Many reports demonstrated that natural polyphenols including anthocyanin also modulate Akt signaling pathways to inactivate the NF-κB, and STAT3 in cancers [38,39,40], but it is not fully elucidated how these natural polyphenols are suppressing these signaling cascades. It is still unknown whether they bind directly or upstream kinases or directly binding key proteins. Therefore, additional studies are required to address these questions.

Secondly, we used an NF-κB stimulator, TNF-α to clearly answer whether the effects of AIMs on CDDP resistance is related to inhibition of NF-κB in MCF-7 cells. The cytokine family, tumor necrosis factor-α (TNF-α), has a versatile function involving many physiological signaling [41]. TNF-α is a potent pleiotropic pro-inflammatory cytokine produced by macrophages, neutrophils, fibroblasts, keratinocytes, NK cells, T and B lymphocytes, and tumor cells [42]. Studies have shown that MCF-7 cells are vulnerable to high doses of TNF-α treatment [43], while some studies revealed that low doses of TNF-α treatment (20 ng/ml) may induce cell proliferation in MCF-7 cells [42]. We initially chose a low dose as an NF-κB inhibitor, but it induced anti-cancer effects on MCF-7 cells. Literature suggests that TNF-α can induce both apoptosis and inhibit apoptosis through NF-κB activation in cancer cells [44,45,46]. In the present study, we showed 20 ng/ml of TNF-α treatment with AIMs, and with or without CDDP, it increased the anti-cancer activities. The use of TNF-α is applicable for the patients with cancer with resistance because the level of TNF-α is highly increased in patients with advanced and metastatic cancer, and it is associated with cancer progression [47,48]. This study showed that TNF-α shows a synergistic effect with AIMs, and it can be used as a therapeutic tool by inhibiting NF-κB activity in platinum drug resistant cancers.

Lastly, the concentration of the AIMs used in this study seemed to be high for in-vivo studies due to toxicity. However, AIMs did not demonstrate toxicity to normal cells, and in vivo studies with different concentrations of AIMs have already been reported [49]. The present study is also in line with the other studies which showed the anti-cancer properties of anthocyanin in vitro [13,50,51].

In conclusion, AIMs enhanced CDDP sensitivity by inhibiting Akt and NF-κB activity of MCF-7 cells that show relative intrinsic CDDP resistance. This study provides evidence that the addition of AIMs to CDDP would an alternative option for a combination of TNF-α inhibitor and CDDP in human breast cancer.

## 4. Material and Methods

### 4.1. Cell Culture and Chemicals

MDA-MB-231 and MCF-7 human breast cancer cell lines from ATCC were cultured in RPMI-1640 (Hyclone, Waltham, MA, USA) with 10% fetal bovine serum heat (FBS, Gibco-BRL, Grand Island, NY, USA) inactivated at 56 °C for 45 min 1 mM L-glutamine, 100 U/ml penicillin, and 100 μg/ml streptomycin at 37 °C, in CO_2_ Incubator. Protein molecular markers were obtained from Geneaid (New Taipei City, Taiwan). Primary antibodies, NF-κB, p- NF-κB, FAS, Akt, p-Akt, Bcl-2, Bax, p53, XIAP, Parp-1 were acquired from Santa-Cruz Biotechnology Inc. (Santa Cruz, CA, USA). IκB, p-IκB were purchased from cell signaling technologies Inc. (Beverley, MA, USA). The β-actin antibody was purchased from Sigma (Beverley, MA, USA). Secondary antibodies (anti-mouse, anti-rabbit, anti-goat) purchased from Bethyl laboratories, enhanced chemiluminescence (ECL) kit was purchased from Amersham (Arlington Heights, IL, USA). The chemicals which are not specified here are purchased from Sigma Chemical Co. (St.Louis, MO, USA).

### 4.2. AIM Preparation

AIMs were extracted from the fruits of Meoru. The well matured Meoru fruits were collected at Jiri Mountain, Republic of Korea. Purification and characterization of AIMs (Anthocyanins In Meoru) were described previously [52]. Briefly, Anthocyanin pigments were extracted by maceration of the fruits (100 g) in methanol containing 0.1% HCl at 5 °C for 24 h. The extraction procedure was repeated three times. After concentration under reduced pressure (Rotavapor R-124, Buchi, Flawil, Switzerland), the extract was diluted with distilled water (100 mL) and partitioned against ethyl acetate (3(100 mL). The water layer containing the pigments was concentrated to 50 mL. The concentrate was purified according to established procedures by means of ethyl acetate/water partitioning and adsorption chromatography on a bed of Amberlite XAD-7 (Sigma, Youngin, Korea). AIM contains the following composition; delphinidin-3,5-diglucoside:cyanidin-3,5-diglucoside:petunidin-3,5-diglucoside:delphinidin-3-glucoside:malvdin-3,5-diglucoside:peonidin-3,5-diglucoside: cyanidin-3-glucoside:petunidin-3-glucoside:peonidin-3- glucoside:malvidin-3-glucoside ¼ 1.0:0.5:3.4:28.1:6.4:6.4:4.2: 22.5:4.9:22.5:5.0:22.6.

### 4.3. Trypan Blue Exclusion Assay

Trypan blue staining is used to identify the number of viable and dead cells. The cells were seeded in 6-well plates with the seeding density of 5 × 10^4^ cells/well. After the indicated amount of drug treatment, the cells were incubated for 48 h at 37 °C in a CO_2_ incubator. The cells were trypsinized and collected with the floating cells. After centrifugation, the cells were dissolved in 50 µL of media. In addition, 50 µL of 0.4% trypan blue (Sigma, Beverley, MA, USA) was added and then counted under a light microscope within 5 min using a hemocytometer. The percentage of viable cells is calculated as (1-(Number of dead cells/Number of total cells)) × 100.

### 4.4. DAPI Staining

The cells were seeded in 12 well plates with a density of 1 × 10^5^ cells/well and grown for 48 h with the indicated drug treatment at 37 °C in a CO_2_ incubator. After treatment, the media were removed and cells were washed with 1 X PBS three times. The cells were fixed with a 4% formaldehyde solution in 4 °C for overnight. After incubation, the cells were washed with 1 X PBS thrice. One microliter of 1 µg/ml DAPI was added to 1 mL of 1 X PBS. The cells were kept at 37 °C for 1 h. After 1 h incubation, the cells were washed with 1 X PBS for three times, and 200 µL of 90% glycerol was added and analyzed under the fluorescent microscope.

### 4.5. Cell Cycle Analysis through Flow Cytometry (PI Staining)

The MCF-7 cells were seeded in 6-well plates with a density of 5 × 10^4^ cells/well and treated with the indicated concentration. After 48 h of treatment, the cells were trypsinized and collected with the floating media in a 15 mL falcon tube and centrifuged at 2000 rpm for 5 min. The supernatant was removed and washed with 5 mL of 1 X PBS twice. The pellet was suspended in 300 µL of 1 X PBS and later added with 700 µL of absolute ethanol. The pellet was shifted to a 1.5 mL Eppendorf tube and fixed for 20 min in −20 °C. After fixing, the cells were centrifuged and the pellet was suspended in 500 µL of 1 X PI containing 50 µg/ml PI in 1 X PBS and 50 µg of RNase A followed by incubation in dark for 20 min. After incubation, flow cytometry analyses were performed by Cytomics FC 500 (Beckman Coulter, Brea, CA, USA). The data were analyzed in CXP Software (Beckman Coulter, Inc., Fullerton, CA, USA).

### 4.6. Apoptosis Analysis through Annexin V and PI Staining

Apoptosis cell detection was performed using FITC Annexin V Apoptosis Detection Kit I (BD Pharmingen, San Diego, CA, USA). As per the manufacturer protocol, after treatment, the cells trypsinized and washed with 1 X PBS were re-suspended in 100 µL of binding buffer and stained with 10 µL of Annexin V-FITC and 10 µL PI. The stained cells were incubated for 30 min in dark. After incubation, FACS (Flow cytometry) analysis was performed using Cytomics FC 500 (Beckman Coulter, Brea, CA, USA). The data were analyzed in CXP Software (Beckman Coulter, Inc., Fullerton, CA, USA).

### 4.7. Western Blot Analysis

MCF-7 breast cancer cell lines were seeded in a 10 cm dish plate with a seeding density of 2.2 × 10^6^ cells/well. AIMs were treated for 48 h with a concentration of 400 µg/ml and CDDP was treated with a concentration of 5 µg/ml. For combined treatment, AIMs were treated 1 h before the treatment of CDDP. For TNF-α treatment, 20 ng was treated 1 h before the treatment of AIMs and then CDDP. After treatment, the cells were collected by the use of cell scraper and then centrifuged at 2000 rpm for 5 min. The supernatant was removed and centrifuged again to remove the excess media. After complete removal of the media, the pellet was suspended in 500 µL of 2 X sample buffer (100 mM of Tris-Cl (pH 6.8), 4% (*w/v*) sodium dodecyl sulphate (SDS), 0.2% (*w/v*) Bromophenol blue and 200 mM of DTT (dithiothreitol). The protein lysates were collected in the 1.5 mL Eppendorf tubes and kept at 100 °C for 10 min. The protein was quantified using Bradford assay. In addition, 30 μg of proteins were resolved in 8–12% SDS-PAGE and followed by the transfer to PVDF (polyvinylidene difluoride) membrane. After transfer, the membranes were blocked with 3% skimmed milk in TBST buffer (Tris-buffered saline containing 1% Tween 20) for 30 min at room temperature and incubated at 4 °C for overnight with primary antibodies. After overnight incubation, the membranes were washed with TBST buffer thrice each wash for about 10 min followed by the incubation with 1:2000 dilution of horseradish peroxidase (HRP)-conjugated secondary antibody for 1 h in room temperature. The membranes were later washed with TBST buffer for three times (10 min/wash) subsequently developed with ECL (electrochemiluminescence) solutions (Bio-Rad Laboratory, Hercules, CA, USA).

### 4.8. Statistical Analysis

All experiments were performed in triplicated and the results were expressed as means ± standard deviation (SD). A Student’s *t*-test using SPSS Version 10.0 for Windows (SPSS, Chicago, IL, USA) is used for two-group comparisons, and for three treatment groups, one-way analysis variance with Newman-Kuels is used to calculate the significance. The synergetic index was calculated as previously reported [20] if $\frac{\mathrm{Percentage}\;\mathrm{of}\;\mathrm{AB}}{\mathrm{Percentage}\;\mathrm{of}\;A\;X\;\mathrm{Percentage}\;\mathrm{of}\;B\;}$ < 1 it denotes synergism, if $\frac{\mathrm{Percentage}\;\mathrm{of}\;\mathrm{AB}}{\mathrm{Percentage}\;\mathrm{of}\;A\;X\;\mathrm{Percentage}\;\mathrm{of}\;B\;}$ =1 it denotes additive effect, if $\frac{\mathrm{Percentage}\;\mathrm{of}\;\mathrm{AB}}{\mathrm{Percentage}\;\mathrm{of}\;A\;X\;\mathrm{Percentage}\;\mathrm{of}\;B\;}$ > 1 it denotes antagonism. In the given equations, A and B is the effect of the drug individually and AB is the combined effect.
